# Effect of a sports mouthguard on the functional range of motion of the spine and the upper body posture in taekwondo

**DOI:** 10.1186/s13102-021-00232-0

**Published:** 2021-01-14

**Authors:** Daniela Ohlendorf, Mara Romdhane, Christoph Lehmann, Sebastian Lehmann, Stefan Kopp, Christian Maurer-Grubinger, Gerhard Oremek, David A. Groneberg, Eileen M. Wanke

**Affiliations:** 1 Faculty of Medical Science, Institute of Occupational Medicine, Social Medicine and Environmental Medicine, Goethe University Frankfurt am Main, Theodor-Stern-Kai 7, Building 9A, 60596 Frankfurt/Main, Germany; 2 School of dentistry, Department of Orthodontics, Goethe University Frankfurt am Main, Theodor-Stern-Kai 7, Building 29, 60596 Frankfurt/Main, Germany

**Keywords:** Taekwondoka, Back scan, sonoSens, Custom-made mouthguard, Ready-made mouthguard

## Abstract

**Background:**

The aim is to investigate to what extent the different oral protections compared to the habitual occlusion affect the upper body posture in statics and during taekwondo-specific movement.

**Methods:**

12 Taekwondoka (5 f/7 m) of German national team were measured by using a 3d back scanner and an ultrasonic distance measuring (upright stand, taekwondo attack and defense movement, two taekwondo specific combinations) in habitual occlusion, with a custom-made and ready-made mouth protection

**Results:**

There are no significant changes in the upper body posture (*p* ≥ 0.05). Depending on the dynamic measurements, different significant reactions of the spinal position were found while wearing the custom made mouthguard or the ready-made mouthguard according to the conducted movement.

**Conclusion:**

The measured changes in dynamic movements are not clinical relevant. Based on the positive responses from the participants, the custom-made mouth protection can be recommended combined with an individual analysis.

## Background

In competitive sports, the primary goal is to achieve high sporting success in competitions. It differs from recreational sport in many aspects: it requires considerably more time, more intensive training and focuses on sporting success. Therefore, the training design is an important factor for success.

In the search for additional innovations or performance-enhancing measures, sports science is concerned with the influence of occlusion on body statics and dynamics [1–5]. Beside the protective function, bite splints are intended to generate a more harmonious posture and thus positively promote sporting performance. This interdisciplinary approach to compensate for temporomandibular dysfunctions (TMD), to alleviate acute pain phases and avoid unphysiological tooth contact on the one hand, and to positively change body posture on the other hand, has been analyzed in different studies [6–17]. As early as 1926 Schwarz [18] discovered that the lower jaw adapts to the head movements. Persons with an Angle class II have a slightly anteriorly inclined posture and those with an Angle class III are more inclined posteriorly [12, 19–22]. Marini et al. [23] also demonstrated that a 2 mm thick composite occlusal blockage causes a minimal change in posture in the frontal plane compared to habitual occlusion and induces a transient increase in the activity of masticatory muscles. After a 14-days follow-up period, the experimental occlusal interference did not significantly influence the body posture.

Further studies have also confirmed differences in body posture, postural control, and walking behavior [24–27]. Moon und Lee [28] also analyzed the same results in their review, according to which occlusion and temporomandibular dysfunction can affect head and jaw muscles as well as postural control. Lai et al. [29] showed that in subjects with TMD, the use of splints to correct this dysfunction resulted in increased jump duration and endurance. In contrast, the data of the control group, whose splint was supposed to cause a dysfunction, deteriorated.

The primary function of splints in sports is the prevention of anterior tooth trauma [30–33] like in sports with frequent tooth damage include boxing, ice hockey, martial arts, skateboarding and mountain biking. All sports involving physical contact have an increased risk of tooth injury, such as boxing, hockey or taekwondo [34–37]. In some of these sports mouthguards are even obligatory according to competition regulations, as in the Far Eastern Martial Art Taekwondo, according to which no participation in a competition is allowed without mouthguards and Taekwondoka are therefore used to wearing mouthguards [38]. In sports such as fieldhockey [39], boxing [40], handball [41] or soccer [42], the influence of mouthguards on posture has already been investigated. In almost all of these studies, it has been shown that wearing an individually produced mouthguard, but also wearing a ready-made mouthguard, can cause minimal - positive/negative - changes, which in turn confirms the functional interdependence. Further studies have also investigated the influence of occlusion on posture and verified or falsified correlations [2, 4, 5, 43–47].

Only Cetin et al. [2] tested the influence of custom-made mouth guards on strength and anaerobic performance of taekwondo athletes and showed a significantly improved peak power and average power in Wingate Anaerobic Test and Hamstring Isokinetic Peak Torque when wearing a custom-made mouth guard. Although taekwondo is a Olympic discipline since Sydney 2000 and the wearing of a custom-made mouth guard is mandatory in competition [32] further results in taekwondo are lacking. The aim of this study is therefore to find out whether, in professional taekwondoka, the supporting and locomotor apparatus can be influenced by two different bite blocks of different manufacture compared to the habitual occlusion position. The previously worn splint will be referred to in the following as a “ready-made mouthguard” (rMG), while the new, individually custom-made mouthguard (iMG) was manufactured in a centric condylar position.

## Methods

### Subjects

In this study12 (5w/7 m) taekwondoka of the German Taekwondo national team participated. All relevant antrompometric characteristics are displayed in Table 1.

**Table 1:** Anthropometric characteristics of the study population

Anthropometric parameter	Data	
Number of subjects and gender	12 (5 f/ 7 m)	
Age (mean ± SD)	18–34 years (23.9 ± 5.1)	
Body height	160–190 cm	
Taekwondo experience	11–22 years	
Weight class	Men	Women
	- 58 kg (*n* = 0)	- 49 kg (*n* = 2)
	- 68 kg (n = 2)	- 57 kg (*n* = 0)
	- 80 kg (n = 3)	- 67 kg (*n* = 1)
	+ 80 kg (*n* = 2)	+ 67 kg (*n* = 2)
Training schedule	3–4 times/day: approx. 1 h athletics training, 2x2h taekwondo specific training, 1 h rehabilitation	

As the team was in the preparation for the world championship. All athletes were at a comparable level of performance. However, the physical appearance is very different, as the athletes fight in different weight classes. Exclusion criteria at the time of measurement were acute injuries of any kind, such as herniated discs, fractures, jaw joint problems, temporomandibular dysfunctions (TMD) or excessive gagging. The participation was voluntary. A written declaration of consent of the test persons was available. Personal data and findings of the test persons were determined on the basis of an anamnesis questionnaire.

An approved ethical application from the Goethe University Frankfurt am Main (No. 48/11) was available for the study.

### Three-dimensional back scan

The back scanner “ABWBodyMapper” of the company ABW GmbH (Frickenhausen/ Germany) is a system for three-dimensional, contactless optical measurement of the body posture based on video raster stereography (VRS). The scanner measures 75 × 245 × 585 mm and records 30 images per sequence, with the highest possible image frequency of 50 images per second. For one measuring sequence 15 images were shot, the maximum frequency is 500 frames/sec with a resolution of 1/100 mm. The manufacturer specifies a measuring error of < 1 mm. To be able to analyze the upper body posture, markers were placed at a total of six anatomical fixed points: VP Vertebra prominens (7th cervical vertebra), AISL lower scapula angle left and right, DL dimples (lumbar pits) left and right, SP sacrum point (beginning of rima ani). This allows rotations in the shoulder and pelvic area as well as the shape of the spine to be analyzed.

### Ultrasonic distance measurement

The sonoSens monitor from the company GefreMed (Chemnitz/Germany) works according to the method of sonometry and determines data based on an ultrasound distance measurement. This detects changes in the movement of individual spinal column sections. The instrument uses an ultrasonic frequency of 250 kHz and a measurement frequency of 10 Hz (12 channels). Spine position changes (measurement error: ±0.4 mm) are obtained by measuring the relative and continuous distances between the 4 × 2 sensor pairs that are attached to the skin of the subject’s back using adhesive pads. The four sensor cables with correspondingly eight ultrasonic sensors are attached in pairs to the skin paravertebrally of the spinal column. The measurements are taken parallel and diagonal to the spine. This allows a change in upper body posture in the frontal and transverse planes (extension, flexion, lateral flexion and torsion) for the cervical (CS), thoracic (TS), and lumbar (LS) spine. The sonoSens monitor is a certified measurement device for medical diagnosis (CE mark of the German Medical Devices Act: CE0118). Moreover, several authors verified the reliability and validity for sonoSens monitor [48, 49].

### Mouthguards

#### Ready-made mouthguard

The players’ own mouthguards were manufactured according to different methods of the treating dentist and are referred to as ready-made mouthguards (rMG) in the following. It did not have an adjusted surface - the current bite position was not recorded. The hold in the mouth is not optimal and breathing is difficult because the splint is fixed by the teeth clenching together.

#### Individually custom-made mouthguard (iMG)

The production of the iMG was carried out in several steps on the test person and in the laboratory. It was specifically made for each test person. To produce the iMG, the upper and lower jaws were first made with a double mix impression and then poured with hard plaster in the laboratory and master models were made. The position of the lower jaw, deflection, mouth opening, chewing movements and also the neutral bite position were recorded by an electronic ultrasound-based recording system (Arcus Digma, Kavo/Girrbach/Germany). This was done by applying a bisacrylate-based bite registration agent (Luxabite/DMG/Germany). The template was placed in the mouth and the mandible fixed for 1 min by the practitioner during the curing phase. In the laboratory, the upper jaw models were placed in the articulator (Protar5B/Kavo/Girrbach Germany) with the skull in mind and the lower jaw was inserted with the help of the registration. Subsequently, the mouthguard was fabricated with the thermoforming device (Biostar/Scheu; Iserlohn/Germany). The soft elastic foil [diameter 3.0 × 125 mm] (Bioplast/Scheu; Iserlohn/Germany) was heated, but before this, undercuts of the model were blocked out with small metal beads. Subsequently, the intercuspidation was adjusted while heating the mouthguard in the articulator by impressions of the opposing jaw (for approx. 30 s). The splint was then processed with special drills until it was finished. It was important to make sure that all edges were rounded and polished. The palatal parts of the mouthguard were shortened towards the edge to prevent irritation of the soft tissue. Above all, speech was not unnecessarily impaired, and the wearing comfort was increased.

### Measurement execution

#### Examination procedure of the three-dimensional back scan

In a darkened room, the subjects were measured dorsally while standing. They first had to completely undress the upper body so that the markers could be glued to the back of the patient at six predefined anatomical points. In order to ensure measurement accuracy, the test persons were instructed to position themselves in front of the scanner at a pre-determined, defined distance of 1.5 m and to look forward for the measurement, to apply equal weight to both feet and to adopt a relaxed, habitual posture. The lower jaw should not be moved.

From then on, the three-dimensional back scan was carried out in a randomized sequence under three different measurement conditions (habitual occlusion, iMG, rMG). Three repeated measurements were recorded for each measurement condition. A break of 1 min was observed between the measurement conditions in order to exclude a possible effect of sensorimotor influences of a previous condition on the next one. In the evaluation of the three-dimensional back scan, the upper body is divided into three segments: (1) the course of the spine, (2) the position of the shoulders and (3) the pelvis.

##### Examination procedure for ultrasound distance measurement

Before placing all sensors on the skin, it hairs are to be removed and the skin degreased with alcohol. The sensors are then fixed to the skin with an elastic adhesive strip such as Fixomull Stretch (Beyersdorf, Hamburg/Germany). The sensors are attached at a distance of 5 cm paravertebrally to the right and left at the following points: C3, the palpable processus spinosus, Th2, the palpable processus spinosus, between Th12 and L1 at the base of the ribs and at the so-called “pelvic dimples” medial to the spina iliaca posterior superior, above the sacroiliac joint.

For the measurements, various reproducible fluid motion sequences, so-called (poomsae), were performed:

- Attacking movement: cut cleavage Chagi and splitting Chagi
- Defensive movement: Forefoot splitting Chagi and paraplegia Chagi
- Movement 1: Paltung Chagi, front foot Dollyeo Chagi
- Movement 2: Paraplegia Chagi and forefoot Dollyeo Chagi

In addition to these leg techniques the first punch Jumog Jireugi was added in movements 1 and 2. The test persons were encouraged to perform the kick exercise with their favourite leg at a kick height in the hip-chest area.

In addition, the stand was recorded.

Here the extension, flexion, lateral flexion and torsion in the cervical, thoracic and lumbar spine area (mm) were used as evaluation parameters.

All measurements were carried out in habitual occlusion and the two mouthguards over a period of 2 min.

### Statistical evaluation procedures and data analysis

The measured values were evaluated with the statistics program BiAS 10.0 (epsilon-Verlag, Norderstedt/Germany). Since the Kolmogorov-Smirnov adjustment test could not prove a normal distribution of the data, only non-parametric tests were used. Accordingly, the Friedman test with subsequent multiple pair comparisons (Conover-Iman test) was used. Subsequently, all *p*-values underwent a Bonferroni-Holm correction. A significance level of *p* ≤ 0.05 was determined for all statistical tests.

## Results

### Results of the three-dimensional back scan

Table 2 shows the mean value and standard deviation of each of the three measurement conditions together with the *p*-value of a comparison of the measurement condition for each parameter. The trunk length D (mm) shows with *p*-value of *p* ≤ 0.05 a significant difference between the measurement conditions. Mean values of 482–489 mm were achieved under all measuring conditions. In a direct pair comparison, a significant p-value of *p* ≤ 0.04 was found between the rMG and the iMG.

**Table 2:** Mean values (MW), standard deviations (SD) and *p*-values of the spine parameters of all measurement conditions in rest position (N), with ready-made mouthguard (rMG) and individual, custom-made mouthguard (iMG). The respective units of measurement are shown at the individual parameter. Significant *p*-values are marked in bold

	Neutral		rMG		iMG		
	MW	SD	MW	SD	MW	SD	*p*-value
**Spine parameter**							
Trunk length D (mm)	489.59	27.30	488.59	26.82	482.36	24.49	0.05
Trunk length S (mm)	527.68	29.88	526.31	27.86	520.27	23.60	0.06
Sagittal trunk decline (°)	−2.99	2.22	−2.65	2.10	−2.63	1.64	0.81
Frontal trunk decline (°)	−0.47	1.33	−0.11	1.16	− 0.42	1.09	0.86
Axis decline (°)	−0.19	2.58	0.04	2.94	−0.09	2.67	0.48
Thoracic bending angle (°)	18.37	3.82	17.75	3.98	18.12	3.63	0.94
Lumbar bending angle (°)	13.58	2.64	13.56	2.16	14.03	2.69	0.46
Standard deviation lateral deviation (mm)	5.40	2.47	5.69	3.13	5.39	2.34	0.62
Standard deviation rotation (°)	2.76	3.51	4.36	2.21	5.50	2.57	0.25
Kyphosis angle (°)	50.96	8.05	51.77	4.77	51.47	6.01	0.25
Lordosis angle (°)	38.95	8.36	36.63	7.55	40.25	8.36	0.69
**Shoulder parameter**							
Scapular distance (mm)	188.81	13.63	186.77	16.41	186.58	16.86	0.84
Scapular height (°)	−2.63	6.34	−5.30	8.70	−5.14	7.55	0.86
Scapular rotation (°)	0.76	4.68	0.78	3.46	1.33	4.08	0.96
Scapular angle left (°)	26.71	6.49	27.07	6.75	26.70	7.27	0.71
Scapula angle right (°)	26.79	9.85	28.19	6.82	24.97	7.89	0.23
**Pelvis parameter**							
Pelvis distance (mm)	104.59	16.56	101.79	14.32	97.16	13.41	0.19
Pelvis height (°)	0.19	2.64	0.15	2.71	0.17	3.20	0.90
Pelvis height (mm)	0.03	4.76	0.21	4.63	0.03	5.14	0.75
Pelvis torsion (°)	−0.06	2.63	1.19	2.52	1.32	2.31	0.27
Pelvis rotation (°)	−0.84	5.34	−0.57	4.00	−0.79	5.29	0.63

Apart from the trunk length D, no other significant values were obtained for the spinal parameters using the Friedman test.

#### Ultrasonic distance measurement

##### Standing

Table 3 shows mean values and standard deviations together with *p*-values of the measurement condition comparison during standing in extension, flexion and lateral flexion as well as torsion. For the left side of the thoracic spine, extension, flexion and lateral flexion show a significance of *p* ≤ 0.01 with values of *N* = 301.25 ± 29.53 mm, rMG = 302.69 ± 35.66 mm, iMG = 314.86 ± 63.81 mm. There are significant differences between the neutral situation in the resting position without mouthguard (N) and the ready-made mouthguard (rMG) in the left thoracic spine with *p* ≤ 0.01 as well as between the rMG and the iMG with *p* ≤ 0.02.

**Table 3:** Mean values (MW), standard deviations (SD) and *p*-values during standing (sonoSens monitor) of all measurement conditions in rest position (N), with ready-made mouthguard (rMG) and individual, custom-made mouthguard (iMG). The respective units of measurement are shown at the individual parameter. Significant *p*-values are marked in bold

	MW			SD			***p***-value
Standing	N	rMG	iMG	N	rMG	iMG	
**Extension/Flexion/Lateralflexion**							
Cervical spine left (mm)	109.14	101.90	102.09	33.64	34.78	33.36	0.17
Cervical spine right (mm)	109.06	99.72	103.49	27.95	22.13	23.40	0.19
Thoracic spine left (mm)	301.25	302.69	314.86	29.53	35.66	63.81	**0.01**
Thoracic spine right (mm)	305.76	304.74	317.19	32.02	40.79	69.70	0.10
Lumbar spine left (mm)	125.92	114.96	123.34	31.31	33.19	33.42	0.08
Lumbar spine right (mm)	150.01	115.25	123.35	98.85	30.55	27.63	0.11
**Torsion**							
Cervical spine left (mm)	117.39	113.39	122.97	25.55	20.39	23.38	0.32
Cervical spine right (mm)	118.61	115.76	114.84	26.38	29.54	28.28	0.54
Thoracic spine left (mm)	348.51	321.27	358.29	89.83	37.15	100.28	**0.04**
Thoracic spine right (mm)	317.67	319.77	320.66	37.30	38.99	68.91	0.20
Lumbar spine left (mm)	144.69	136.68	153.32	27.00	29.64	30.77	**0.01**
Lumbar spine right (mm)	145.84	145.90	150.59	23.77	29.41	30.96	0.32

In torsion, the left thoracic spine (*p* ≤ 0.04; *N* = 348.51 mm ± 89.83, rMG = 321.27 mm ± 37.15; iMG = 358.28 mm ± 100.28) and the left lumbar spine (*p* ≤ 0.02; *N* = 144.69 mm ± 27.00; rMG = 136.86 mm ± 29.64; iMG = 153.32 mm ± 30.69) are significant. While the pair comparison for the left thoracic spine shows no significance, there are differences in the left lumbar spine between N and rMG (*p* ≤ 0.02) and rMG and iMG (*p* ≤ 0.01).

#### Attack and defence

Table 4 shows the mean values, standard deviations and *p*-values of the Friedman test of attack and defense.

**Table 4:** Mean values (MW) and standard deviations (SD) of the attack and defense in rest position (N), with the ready-made mouthguard (kMG) and individual, custom-made mouthguard (iMG). Significant *p*-values are marked in bold

	MW			SD			***p***-value
Attack	N	rMG	iMG	N	rMG	iMG	
**Extension/Flexion/Lateralflexion**							
Cervical spine left (mm)	111.13	107.35	100.49	30.60	35.67	34.63	0.16
Cervical spine right (mm)	110.11	103.98	102.16	25.84	24.10	33.12	0.23
Thoracic spine left (mm)	303.54	299.70	349.70	33.85	39.40	93.88	**0.01**
Thoracic spine right (mm)	304.28	301.80	311.54	32.38	41.15	46.09	**0.04**
Lumbar spine left (mm)	140.54	112.89	122.51	59.07	30.83	32.20	0.10
Lumbar spine right (mm)	132.87	113.77	125.42	42.20	29.37	29.47	**0.03**
**Torsion**							
Cervical spine left (mm)	120.96	114.82	122.04	26.58	22.14	23.10	0.14
Cervical spine right (mm)	121.83	117.39	115.42	29.41	31.44	22.55	0.51
Thoracic spine left (mm)	344.14	318.81	352.43	84.01	39.25	89.36	**0.01**
Thoracic spine right (mm)	327.54	316.63	348.32	52.00	40.56	82.53	**0.001**
Lumbar spine left (mm)	142.81	135.34	145.97	27.08	28.09	25.08	**0.01**
Lumbar spine right (mm)	144.59	146.31	151.63	26.80	30.18	26.94	0.05
Defense							
**Extension/Flexion/Lateralflexion**							
Cervical spine left (mm)	113.17	103.61	108.78	32.42	37.95	36.83	0.08
Cervical spine right (mm)	109.82	99.48	109.69	24.45	27.43	26.73	0.01
Thoracic spine left (mm)	305.77	301.63	317.97	35.07	36.51	58.64	0.02
Thoracic spine right (mm)	304.77	303.51	310.84	35.20	40.45	48.38	0.01
Lumbar spine left (mm)	123.24	116.87	115.24	33.14	32.05	30.96	0.71
Lumbar spine right (mm)	125.55	117.26	116.75	30.75	30.93	26.05	0.54
**Torsion**							
Cervical spine left (mm)	121.69	114.08	122.00	25.02	24.98	18.37	0.01
Cervical spine right (mm)	123.61	117.38	118.49	27.78	31.41	25.09	0.19
Thoracic spine left (mm)	314.34	320.01	352.79	82.45	37.97	85.30	0.01
Thoracic spine right (mm)	325.01	318.72	331.91	47.15	38.83	53.00	0.01
Lumbar spine left (mm)	144.75	137.99	139.29	28.48	29.53	26.08	0.32
Lumbar spine right (mm)	144.94	147.77	148.29	26.61	31.05	27.80	0.54

In attack, there was a significant difference (*p* ≤ 0.01) in extension, flexion and lateral flexion directions for the left side of the thoracic spine (*N* = 303.54 ± 33.85 mm, rMG = 299.70 ± 39.40 mm, iMG = 349.70 ± 93.88 mm). The same was true for the right thoracic spine with *p* ≤ 0.04 (*N* = 304.28 ± 32.38 mm; rMG = 301.80 ± 41.15 mm; iMG = 311.54 ± 46.09 mm). A *p* value of 0.03 was present in the right lumbar spine (*N* = 132.87 ± 42.20 mm; rMG = 113.42 ± 29.37 mm; iMG = 125.42 ± 29.47 mm).

For the torsional position during the attack position, significant values were found in the left (*p* ≤ 0.01) and right thoracic spine (*p* ≤ 0.001) and in the left lumbar spine (*p* ≤ 0.01). The mean values and standard deviations in the range of the left thoracic spine varied between *N* = 344.14 ± 84.0 mm; rMG = 318.81 ± 39.25 mm and iMG = 352.43 ± 89.36 mm. For the right thoracic spine, MW was obtained for *N* = 327.54 ± 52.00 mm, rMG = 316.63 ± 40.46 mm and iMG = 348.32 ± 82.53 mm. In the left lumbar spine the following values were recorded: *N* = 142.38 ± 27.08 mm, rMG = 135.34 ± 28.09 mm and iMG = 145.97 ± 25.08 mm.

In the defensive, the extension, flexion and lateral flexion directions of the right cervical spine (*p* ≤ 0.01; *N* = 109.82 ± 24.45 mm, rMG = 99.48 ± 27.43 mm, iMG = 109.68 ± 26.73 mm), the right thoracic spine (*p* ≤ 0.01; *N* = 304.77 ± 35.20 mm, rMG = 303.51 ± 40.45 mm, iMG = 310.84 ± 48.38 mm) and in the left thoracic spine (*p* ≤ 0.02; *N* = 305.77 ± 35.07 mm, rMG = 301.63 ± 36.51 mm, iMG = 317.97 ± 58.64 mm) significantly differed (Table 3).

In the torsional position there were significant differences in the left cervical spine (*p* ≤ 0.01; *N* = 121.69 ± 25.02 mm, rMG = 114.08 ± 24.98 mm, iMG = 122.00 ± 18.37 mm), in the left thoracic spine (*p* ≤ 0.01; *N* = 314.34 ± 82.45 mm; rMG = 320.01 ± 37.97 mm, iMG = 352.79 ± 85.30 mm) and in the right thoracic spine (*p* ≤ 0.01; *N* = 325.01 ± 47.15 mm, rMG = 318.72 ± 38.83 mm, iMG = 331.91 ± 53.00 mm).

Table 5 shows the corrected pair comparison *p*-values during attack and defense. During attack, differences between the rMG and the iMG could be seen in the left thoracic spine (*p* ≤ 0.001), the right thoracic spine (*p* ≤ 0.02) and lumbar spine (*p* ≤ 0.02). In torsion, the comparison between rMG and iMG of the left lumbar spine (*p* ≤ 0.03) was significant.

**Table 5:** Bonferroni Holm corrected p-values of the pair comparison during attack and defense for the movement directions extension/flexion/lateral flexion and torsion

Segment	Comparison	***p***-value	
**Attack**		Ex/Flex/Lat	Torsion
Thoracic spine left	rMG vs. iMG	0.001	0.01
Thoracic spine right	rMG vs. iMG	0.02	0.001
Lumbar spine right	rMG vs. iMG	0.02	
Lumbar spine left	rMG vs. iMG		0.03
**Defensive**			
Cervical spine right	N vs. rMG	0.01	
Cervical spine right	N vs. rMG / rMG vs. iMG		0.001/0.001
Thoracic spine left	N vs. rMG	0.02	
Thoracic spine left	rMG vs. iMG	0.02	0.01
Thoracic spine right	rMG vs. iMG	0.01	0.01

In the defensive position there were differences between the resting position and the rMG in the right cervical spine (*p* ≤ 0.01). Differences between the resting position and the iMG or the rMG and the iMG in the left thoracic spine confirmed a p-value of 0.02 in each case. On the right side of the thoracic spine, however, there was a difference between rMG and iMG (*p* ≤ 0.01). In the case of torsion, there were significances between the rMG and the iMG in the left cervical spine (*p* ≤ 0.001), the left GVA (*p* ≤ 0.01) and the right thoracic spine (*p* ≤ 0.01). Furthermore, the comparison between the rest position and the rMG was significant (*p* ≤ 0.001).

##### Movement changes of attack and defensive movement

*Attack movement:* The cervical spine position was symmetrical, with the thoracic spine slightly inclined to the left but rotated to the right. In contrast to this position, the lumbar spine was inclined to the right combined with a left rotation.

In comparison to the neutral occlusion position, there were significant changes in the taekwondo attack position between the rMG and the iMG in the area of the left and right thoracic spine. While the thoracic spine extension was more pronounced when the rMG is worn, the iMG increases flexion, right lateral flexion and right lateral torsion. In addition, the lumbar spinal flexion position increased with reduced right-sided torsion due to the iMG.

*Defensive movement:* In the average posture, a tendency towards right-sided inclination and rotation could be observed in the cervical spine. In the area of the thoracic spine, there was a minimal right inclination with left-sided rotation components. In the lumbar spine, lateral flexion to the left was evident. The cervical spine was extended by the ready-made mouthguard in combination with a right lateral flexion and rotation. In the thoracic spine area, an extension and lateral flexion to the left was performed. The individual mouthguard caused a lateral flexion to the right and a rotation to the right in the thoracic spine area. Furthermore, an increased pressure load on the right toe area could be observed. The comparison of the two mouthguards showed that the rMG has caused an extension of the cervical spine and flexion in the thoracic spinal region, which resulted in significant torsional stress, but the extent of which has remained the same.

#### Movement I and II

##### Movement I

In extension/flexion/lateral flexion, significant differences were observed in the left thoracic spine (*p* ≤ 0.02; *N* = 302.80 ± 34.47 mm, rMG = 299.73 ± 35.76 mm, iMG = 304.29 ± 41.84 mm) and right thoracic spine (*p* ≤ 0.01; *N* = 303.19 ± 34.94 mm, rMG = 302.02 ± 40.07 mm, iMG = 303.39 ± 45.62 mm). In torsional position the left cervical spine (*p* ≤ 0.03; *N* = 118.31 ± 23.10 mm, rMG = 112.59 ± 24.37 mm, iMG = 117.00 ± 24.11 mm), the left thoracic spine (*p* ≤ 0.01; *N* = 328.24 ± 50.43 mm, rMG = 318.14 ± 35.72 mm and iMG = 336.97 ± 52.74 mm) as well as the right thoracic spine (*p* ≤ 0.01; *N* = 319.93 ± 37.89 mm, rMG = 317.17 ± 37.89 mm, iMG = 344.02 ± 79.09 mm) showed significant differences (Table 5).

##### Movement II

Significances in extension, flexion or lateral flexion were only visible in the right thoracic spine (*p* ≤ 0.03) at values of *N* = 299.28 ± 35.17 mm, rMG = 319.06 ± 90.92 mm and iMG = 281.66 ± 95.42 mm. During the torsional position, the left cervical spine (*p* ≤ 0.04; *N* = 116.12 ± 22.72 mm, rMG = 113.29 ± 21.31 mm, iMG = 114.47 ± 22.93), the left thoracic spine (*p* ≤ 0.02; *N* = 316.75 ± 35.96 mm, rMG = 335.05 ± 86.34 mm, iMG = 326.92 ± 29.83 mm) and in the right thoracic spine (*p* ≤ 0.01; *N* = 313.52 ± 36.70 mm, rMG = 332.84 ± 86.67 mm, iMG = 322.84 ± 42.37 mm) (Table 6) show significance differences.

**Table 6:** Mean values (MW) and standard deviations (SD) of the movement sequence I and II in rest position (N), with the ready-made mouthguard (kMG) and individual, custom-made mouthguard (iMG). Significant *p*-values are marked in bold

	MW			SD			***p***-value
Movement sequence I	N	rMG	iMG	N	rMG	iMG	
**Extension/Flexion/Lateralflexion**							
Cervical spine left (mm)	109.33	101.72	98.10	30.70	37.70	29.09	0.09
Cervical spine right (mm)	105.68	96.76	102.96	22.19	25.78	22.43	0.10
Thoracic spine left (mm)	302.80	299.73	304.29	34.47	35.76	41.84	0.02
Thoracic spine right (mm)	303.19	302.02	303.39	34.94	40.07	45.62	0.01
Lumbar spine left (mm)	124.66	117.47	136.58	34.32	32.22	88.14	0.25
Lumbar spine right (mm)	127.19	119.14	132.65	32.46	31.65	68.13	0.54
**Torsion**							
Cervical spine left (mm)	118.31	112.59	117.00	23.10	24.37	24.11	0.03
Cervical spine right (mm)	121.18	115.66	115.09	27.80	32.27	25.44	0.16
Thoracic spine left (mm)	328.24	318.14	336.97	50.43	35.72	52.74	0.01
Thoracic spine right (mm)	319.93	317.17	344.02	41.25	37.89	79.09	0.01
Lumbar spine left (mm)	145.41	139.20	138.08	29.62	28.88	28.97	0.28
Lumbar spine right (mm)	146.42	149.95	147.16	27.99	31.14	30.12	0.77
**Movement sequence II**							
**Extension/Flexion/Lateralflexion**							
Cervical spine left (mm)	107.82	101.41	96.55	30.95	26.08	27.48	0.25
Cervical spine right (mm)	103.17	101.05	98.05	21.42	22.35	22.91	0.71
Thoracic spine left (mm)	298.23	317.14	302.18	32.33	83.62	38.21	0.32
Thoracic spine right (mm)	299.28	319.06	281.66	35.17	90.92	95.42	0.03
Lumbar spine left (mm)	124.27	132.72	115.24	33.09	55.63	30.65	0.71
Lumbar spine right (mm)	126.26	132.02	116.17	31.56	49.64	27.59	0.77
**Torsion**							
Cervical spine left (mm)	116.12	113.29	114.47	22.73	21.31	22.93	0.04
Cervical spine right (mm)	119.86	119.15	112.37	27.81	19.90	23.50	0.32
Thoracic spine left (mm)	316.75	335.05	326.92	35.96	86.34	29.83	0.02
Thoracic spine right (mm)	313.52	332.84	322.40	36.70	86.67	42.37	0.01
Lumbar spine left (mm)	144.73	139.63	136.83	29.30	29.4	27.21	0.46
Lumbar spine right (mm)	146.13	146.61	145.85	28.24	32.91	29.39	0.54

A pair comparison of movement I (Table 7) showed that the left thoracic spine was significant for the comparison between the rest position and the rMG (*p* ≤ 0.02) and between the rest position and the iMG (*p* ≤ 0.02). The right thoracic spine had a *p*-value of 0.01 in the comparison between rMG and iMG. In torsion, the comparison between the rest position and the rMG in the left cervical spine (*p* ≤ 0.03) and between the rMG and iMG (*p* ≤ 0.01) in left and right thoracic spine differed significantly.

**Table 7:** Bonferroni Holm corrected *p*-values of the pair comparison during movement sequence I and II for the movement directions extension/flexion/lateral flexion and torsion

	Comparison	***p***-value	
**Movement sequence I**		Ex/Flex/Lat	Torsion
Cervical spine left	N vs. rMG		0.03
Thoracic spine left	N vs. rMG / N vs. iMG	0.02/0.02	- / 0.01
Thoracic spine right	kMS vs. iMG / N vs. iMG	0.01	0.01
**Movement sequence II**			
Thoracic spine right	rMG vs. iMG / N vs. rMG	0.03 / -	0.01/ 0.04
Thoracic spine left	rMG vs. iMG		0.01

The pair comparison of movement II (Table 5) showed differences between the rMG and the iMG in the right thoracic spine (*p* ≤ 0.03) in flexion/extension/lateral felxion and torsion. With regard to torsion, the comparison between N and rMG was also significant (*p* ≤ 0.04). For the left thoracic spine, the comparison between the rMG and the iMG was significant (*p* ≤ 0.01).

##### Movement I

The posture in the Taekwondo combination movement 1 had a cervical spine position that was tilted and twisted to the right when at rest. The thoracic spine was slightly rotated to the left, whereas the lumbar spine was inclined to the left. The comparison between the neutral occlusion position and wearing an occlusion aid lead to the following findings: Wearing the iMG resulted in right lateral flexion and left rotation in the area of thoracic spine. The rMG, on the other hand, lead to a thoracic spine extension, which was manifested as a change in thoracic spine rotation.

##### Movement II

Also in the Taekwondo combination movement 2 the cervical spine is tilted and rotated to the right. The thoracic spine was rotated compensatory to the left. In the area of the lumbar spine, a left-sided lateral flexion and right-sided rotation could be seen.

Wearing the rMG opposite to the resting position results in a thoracic spine flexion, which manifests itself in a significance of the torsion of this area, whereby the descriptive parameters remained unchanged.

The comparison of both mouthguards revealed an increased thoracic spine extension due to the iMG, which had a significant effect on an increased left-sided rotation.

## Discussion

This study was based on the question whether temporarily caused occlusal changes in the upper body posture of competitive athletes in Taekwondo can be detected in statics and dynamics. For this purpose, the measurement condition at rest of the lower jaw was considered as a reference measurement, from which the respective deviations of the other two measurement conditions (iMG, rMG) are listed.

With regard to the body posture in the resting position of the mandible, the upper body of the test persons is almost symmetrical and only inclined approx. 3° ventrally. The upper body deviated marginally to the left, which lead to a slight compensatory counter-movement of the spinal column to the right. Furthermore, the thoracic kyphosis was about 12° more pronounced than the lumbar lordosis. In addition, the left scapula was approx. 2.7° more cranial and 0.76° more ventral, with both shoulder angles being almost equal. The right shoulder blade was positioned further cranially and rotated and twisted ventrally. The ultrasonic distance measurement corresponded to the same description as the three-dimensional back scan. In this respect, the data from the Taekwondoka correspond to those of young, healthy men and women between 18 (20) and 35 (30) years of age who were also examined with the back scanner [50, 51]. The only difference is the slightly larger thoracic bending angle of the athletes. Both the young healthy men [51] and women [50] and the present competitive athletes showed symmetrical body posture. The comparison of the upper body posture with those of professional male field hockey players shows that they also have a quite symmetrical posture, apart from the pelvic position. The pelvis of field hockey players is on the right side further cranially and rotated dosally as well as twisted to the right [52].

When wearing the individual custom-made mouthguard (iMG) temporarily, only the trunk length is significantly reduced by approx. 7 mm. With regard to ultrasound distance measurement, both occlusal splints lead to a more inflected left thoracic spine with more symmetrical left lateral flexion. In addition, left-sided torsion increased in the left lumbar spine area. Descriptive observation of the static evaluation parameters revealed that wearing both splints causes only slight angular or millimeter deviations from the rest position. Furthermore, most deviations of the back scan were within the range of ±1° or mm according to the manufacturer’s specifications. Also the deviations of the ultrasonic distance measurement do not exceed a difference of 5% and therefore had no real clinical relevance. The fact that occlusal influences had no influence on the body posture in competitive athletes of different disciplines has also been proven in other studies [53, 54]. In contrast, it could be seen in male field hockey players that both mouthguards cause negative upper body changes when standing compared to the neutral measurement in habitual bite position without mouthguard. When comparing the two mouthguards, the individually manufactured mouthguard caused less negative effects than the ready-made mouthguard [52].

When analyzing the dynamic measurement conditions, it must be taken into account that the musculature is constantly active and must adapt to new conditions as quickly as possible. The four motion sequences reconstructed from the measurement data are similar to the motion description in the textbook [55]. If a changed occlusion position - in the form of the individually or ready-made mouthguard - is added during these same movement sequences, statistically significant effects in the area of the spinal column become apparent. No significant differences could be seen between the two mouthguards rMG and iMG. However, since these significances were only minor based on clinical interpretation, we assume that these effects can only be considered as trends and less as generalizable changes. Therefore, we conclude that functional interdependencies between the jaw position and the musculoskeletal system do exist, but they must always be examined subjectively with regard to their effects. Furthermore, we suspect that the bite change (rMG, iMG) caused has an effect on the functioning of the surrounding musculature as well as on the masticatory muscles (M. masseter, *M. temporalis* und Mm. pterygoidei lateralis und medialis) and on the temporomandibular system. The occlusion of the teeth of the maxilla and mandible permanently adapt to the head movement [56]. In female field hockey players, it has been shown that wearing a mouthguard (rMG, iMG) caused changes in hockey-specific movements, especially in the thoracic spine. Especially the iMG lead to a derotated and extended posture [39]. Similar results have been observed with boxers [40]. Here, too, the comparison of the different occlusal conditions (iMG, rMG, rest position) in statics or dynamics showed an extension and reduced rotational movement of the upper body in the cervical spine and thoracic spine area by the iMG. Changes in the plantar pressure distribution were also observed by the iMG. While a slight load change from the rearfoot to the forefoot has occurred during standing, a shift of weight towards the rearfoot is evident during offensive movements. These investigations were both performed with the same research design.

Unconsciously, the head is always held exactly so that the teeth fit together best, so that by influencing the position of the head, the spinal position is influenced and the posture is adjusted [57, 58]. Basically, the explanation of these functional relationships follows from various theoretical constructs, such as the muscle chain and fascial chain theory [59–65], Furthermore, other aspects such as malfunctions in the temporomandibular system, such as cross bite, progeny or distal/buccal occlusion, or injuries of the movement system (acute or chronic) of the respective athletes must also be taken into account in future analyses. It would also be worth paying attention to problems of the temporomandibular system (TMS) in martial arts athletes and the related problems, such as headaches. These malfunctions or problems of the TMS can also trigger that a mouthguard can cause more far-reaching, positive or negative changes. In order to better assess the effects of such factors, however, data from healthy athletes without such problems are first of all relevant, so that a better classification of the changes can then be made in addition to a comparison.

The present study has come to similar conclusions as Collares et al. [3], Golem et al. [5] and Arent et al. [1]. Regardless of the evaluation parameter or measuring method examined, all of them came to the conclusion that an individually produced mouthguard has no positive effect on the measured parameters, but also does not cause any negative effects. The performance of the athlete was not impaired. A further aspect that makes it difficult to compare the results with those of other investigation is based on the fact that the dynamic measurements were carried out using movement sequences. This has not been done to this extent in other studies of other research groups to date when occlusal effects on dynamics have been investigated. Most of these studies have only been carried out while running or walking, mainly on the treadmill [66, 67]. The faster the walking speed, the stronger the activity of the M. masseter [68]. However, no significant differences have been measured between an individual mouthguard and a ready-made mouthguard in taekwondoka [2].

Whether or not a permanent occlusal change has a long-term effect on posture, Saito et al. [69] have shown that patients with an anteriorenal disc displacement had a head deviation to the right and a lordosis of the spinal column compared to a healthy control group. Although this study [69] confirms the correlation between upper body posture and the temporomandibular system, it cannot be compared with the present study, as the sports mouthguards only provoked a temporary and not a permanent occlusal change. Furthermore, it has not yet been proven whether the long-term wearing of a sports mouthguard can cause these changes.

We conclude that it is possible that the compensation mechanisms of conscious and unconscious body reactions are more pronounced in standing than in dynamics. Further, we consider that of greater importance for competitive athletes, however, are the findings from the dynamic examination phases. In this respect, it should be noted that the above-average muscle development of athletes compared to amateur athletes or non-athletes leads to a better neurophysiological interception or compensation of external influences. The pronounced support and holding musculature stabilizes the joints and fluctuations are thus compensated [70]. The selection of these athletes proved to be very difficult, since on the one hand many training and competition sessions took place and on the other hand injuries limited the complete participation in this examination. The already small circle of potential participants was additionally reduced by gagging (during impression taking). A further exclusion criterion was temporomandibular dysfunction. Future analyses may include additional cofactors that may affect occlusal changes in Taekwondoka. These include age, gender or weight class during competition. The latter would have to be analyzed under the aspect whether a different fighting weight also includes a different fighting style or fighting movements. However, since the selection of professional Taekwondoka is very small, this implementation turns out to be very difficult. Therefore, this subpopulation analysis would be more feasible with non-professional taekwondoka.

Another interesting question, which arises from the present results, is whether the centric condyle position brought about by the iMG would lead to a significant change in upper body statics and dynamics during long-term use (longer than 12 months). Also the aspect of whether the intensity of the training and the wearing period of the mouthguard cause different effects cannot be answered to date. Furthermore, it cannot be clarified in this study whether the high training volume and the associated high physical strain are the cause of the low effects of mouthguards on static and dynamic upper body posture. Therefore, it would be interesting to compare the following results with Taekwondoka, whose training volume is lower or with non-athletes. The comparison with the latter seems problematic, because non-athletes would not be able to perform the given movement sequences in a technically correct way.

The constitution of the participants in the present study has been very different, as they fought in all weight classes between 50 kg and 90 kg. However, since they are competitive athletes of the German national team, these criteria are more difficult. The two biggest influencing factors are the high fluctuation of athletes due to performance and/or injury, so that many athletes only stay with the national team for a short time. This includes both male and female teakwondoka. It is precisely for this reason that both genders were included in the analysis. Since no gender-specific differences in the assumed neurophysiological reactions have been scientifically proven to date, we assume that they are identical.

Summarizing, an individual sports mouthguard is recommended in any case, because the biting with the splint brings the jaw joints into a central condylar position, whereas the ready-made mouthguard leads the athlete into a habitual position. In this position, the three-dimensional allocation of the lower jaw to the upper jaw is not taken into account when manufacturing the mouthguard. While they bite to the maximum, this leads to an increase in strength, since the individual mouthguard brings the jaw joint into a central, balanced condylar position on both sides of the jaw.

## Conclusion

Due to the low impact of wearing an individually manufactured or ready-made mouthguard on the upper body posture in terms of statics and dynamics, it is recommended to carry out an individual analysis of each athlete, especially with regard to a possible temporomandibular dysfunction. Furthermore, it should be noted that the individual mouthguard does not have any negative effect and therefore does not negatively affect the athletes’ performance in any way. Competitive athletes can only benefit from an individually manufactured mouthguard.

## Data Availability

The datasets used and/or analysed during the current study are available from the corresponding author on reasonable request.
